# Pathogenic copy number variants that affect gene expression contribute to genomic burden in cerebral palsy

**DOI:** 10.1038/s41525-018-0073-4

**Published:** 2018-12-14

**Authors:** Mark A. Corbett, Clare L. van Eyk, Dani L. Webber, Stephen J. Bent, Morgan Newman, Kelly Harper, Jesia G. Berry, Dimitar N. Azmanov, Karen J. Woodward, Alison E. Gardner, Jennie Slee, Luís A. Pérez-Jurado, Alastair H. MacLennan, Jozef Gecz

**Affiliations:** 1 Robinson Research Institute & Adelaide Medical School, University of Adelaide, Adelaide, South Australia 5000 Australia; 2 Data61, Commonwealth Scientific and Industrial Research Organisation, Ecosciences Precinct, Dutton Park, Brisbane, QLD 4102 Australia; 3 School of Biological Sciences, University of Adelaide, Adelaide, South Australia 5005 Australia; 4 Department of Diagnostic Genomics, Queen Elizabeth II Medical Centre, PathWest, Nedlands, WA 6009 Australia; 5 School of Biomedical Sciences, University of Western Australia, Perth, WA 6009 Australia; 6 Genetic Services of Western Australia, King Edward Memorial Hospital, Subiaco, WA 6008 Australia; 7 Genetics Unit, Universitat Pompeu Fabra, Barcelona, 08003 Spain; 8 Hospital del Mar Research Institute (IMIM) and Centro de Investigación Biomédica en Red de Enfermedades Raras (CIBERER), Barcelona, 08003 Spain; 9 SA Clinical Genetics, Women’s and Children’s Hospital & University of Adelaide, Adelaide, South Australia 5006 Australia; 10 South Australian Health and Medical Research Institute, Adelaide, South Australia 5000 Australia

**Keywords:** Next-generation sequencing, Neurodevelopmental disorders, Gene expression, Structural variation

## Abstract

Cerebral palsy (CP) is the most frequent movement disorder of childhood affecting 1 in 500 live births in developed countries. We previously identified likely pathogenic de novo or inherited single nucleotide variants (SNV) in 14% (14/98) of trios by exome sequencing and a further 5% (9/182) from evidence of outlier gene expression using RNA sequencing. Here, we detected copy number variants (CNV) from exomes of 186 unrelated individuals with CP (including our original 98 trios) using the CoNIFER algorithm. CNV were validated with Illumina 850 K SNP arrays and compared with RNA-Seq outlier gene expression analysis from lymphoblastoid cell lines (LCL). Gene expression was highly correlated with gene dosage effect. We resolved an additional 3.7% (7/186) of this cohort with pathogenic or likely pathogenic CNV while a further 7.7% (14/186) had CNV of uncertain significance. We identified recurrent genomic rearrangements previously associated with CP due to 2p25.3 deletion, 22q11.2 deletions and duplications and Xp monosomy. We also discovered a deletion of a single gene, *PDCD6IP*, and performed additional zebrafish model studies to support its single allele loss in CP aetiology. Combined SNV and CNV analysis revealed pathogenic and likely pathogenic variants in 22.7% of unselected individuals with CP.

## Neurodevelopment: Copy number variants underlie cases of cerebral palsy

At least 23% of cerebral palsy has a genetic basis, due to either point mutations or large chromosomal abnormalities. Using sequence data from 186 unrelated patients, a team led by Mark Corbett from the University of Adelaide, Australia, searched the protein-coding portion of the genome for disease-causing duplications or deletions, types of mutations collectively known as copy number variants, or CNVs. The researchers singled out 7 pathogenic CNVs that they corroborated through microarray profiling and gene expression analyses. Using a zebrafish model, the researchers identified a new gene “PDCD6IP” associated with cerebral palsy and epilepsy. They also flagged another 14 CNVs that were likely pathogenic but could not be confirmed. The work highlights the need for comprehensive genetic testing to be considered early in the diagnosis of cerebral palsy.

## Introduction

Cerebral palsy (CP) describes a group of heterogeneous disorders affecting movement and posture that are caused by a non-progressive lesion or abnormality in the immature pre or postnatal brain. Individuals living with CP frequently experience additional comorbidities such as mild, moderate or severe intellectual disability (ID), (48–51%), epilepsy (32–35%), speech impairments (60.7%), vision impairments (35.8–40%) and autism spectrum disorders (ASD), (8.9%).^1^ The most common correlated risk factors for CP are prematurity, congenital anomalies and intrauterine growth restriction.

There is growing evidence for a significant contribution of genetics to the aetiology of CP. Familial studies have implicated *GAD1*, *ADD3* and *KANK1* in CP.^2–4^ Genetic studies into some families originally diagnosed with CP have now defined a unique sub-syndrome associated with ID and microcephaly caused by recessive mutations in the genes that comprise the adaptor protein complex 4 (*AP4M1*, *AP4E1*, *AP4S1* and *AP4B1*).^5–7^ We identified pathogenic and likely pathogenic de novo and inherited single nucleotide variants (SNV) in at least 14% of individuals living with CP from an unselected cohort of 98 parent and proband trios.^8^ In a similar fashion, de novo and inherited copy number variants (CNV) have been detected in 31% of cryptogenic CP cases with no identified aetiology, 9.6% in an unselected CP cohort when strict criteria for clinical relevance were used and 23.7% in a cohort of 97 individuals with hemiplegia.^9–11^ Our previous analysis of a cohort of 50 unselected individuals revealed inherited CNV in 20%, however the clinical significance of these was uncertain.^12^ These data collectively make a strong case for significant involvement of genetics in CP.

Whole exome and whole genome sequencing are rapidly becoming the gold standard to diagnose neurodevelopmental disorders. As data accumulate, it becomes possible to reanalyse rare variants with increasing power and for new genes to be implicated in these disorders.^13^ Furthermore, combined analysis of genome and transcriptome data can be used to interpret the effects of DNA variants on gene expression and identify potentially pathogenic coding and non-coding variants.^14^ For an unselected CP cohort of 182 individuals including the 98 trios we previously analysed, we identified an additional nine potentially pathogenic SNV (a further 5% of the cohort) with functional effects on gene expression using this strategy.^15^ We hypothesised that within our wealth of sequence data from individuals living with CP, where our initial focus had been to identify rare coding sequence variants that we would also detect previously unnoticed CNV.

## Results

We had previously analysed these exome data for causative SNV.^8^ Except for a pilot group of 50 cases studied by chromosomal microarray,^12^ copy number analysis was not performed thus the results presented here are novel. Within the analysed cohort of 186 CP cases and parents where available comprising 460 individuals we detected 8136 CNV events ranging in size from 1 kb to over 10 Mb (Fig. S1A). There were ten families included in this study in whom we had previously detected 14 CNV of potential clinical significance using array based detection.^12^ Copy Number Inference From Exome Reads (CoNIFER) analysis detected 11/14 of these CNV; thus sensitivity for detection was an estimated 78% [95% confidence interval (CI), 52.4% to 92.4%] similar to that reported in the original CoNIFER publication.^16^ The three events that were not detected were less than 10 kb in size, each covering single exons (Fig. S1B). We did not detect any additional CNV in these 50 cases that were previously analysed by microarray. We additionally detected nine deletions and 14 duplications in 21 out of 186 probands after filtering (Table 1). Of these CNV, eight were unequivocally assigned as pathogenic because they overlapped significantly with loci that were implicated in known syndromes, one was deemed to be likely pathogenic based on supporting evidence and the remaining 14 were of uncertain pathological significance (Table 1; Tables S1–S23). There were two individuals in whom two CNV were identified. In both cases, these terminal deletion and duplication events resulted from de novo unbalanced reciprocal translocations, validated by karyotyping in the proband and ruled out in both parents (Table 1: 150432P and 181463P).

**Table 1:** CNV identified from exome sequencing

Sample	Sex	CP subtype	GMFCS	Gestation (weeks + days)	Additional phenotypes	Maternal	Position (Cytoband) *Inheritance*	Type	Pathogenicity prediction and known phenotypes
67067P Trio	F	D	3	27 + 4	BW: 89th percentile. PVL, NICU stay, central sleep apnoea (moderate/severe), asthma, home oxygen, hypermetropic astigmatism, hypogammaglobulinaemia, lumbar scoliosis (mild), small infraorbital telangiectasia, good cognition	IVF pregnancy, history of stillbirth, preterm labour, difficult extraction, obstetrical bleeding (coagulopathy), foetal distress, emergency caesarean delivery	1:145112323-149201987 (1q21.1) De novo	Dup	**Pathogenic**. ASD, ID, ADHD, developmental coordination disorder, delayed speech, macrocephaly, cardiac problems, scoliosis, hypotonia, abnormal gait and/or agility.^27^
181463P Duo	F	Q	4	37 + 5	BW: 7th percentile. IUGR, NICU stay. CNV events in this individual result from a de novo unbalanced translocation	Fetal distress, emergency caesarean without onset of labour, decreased fetal movements from 32 weeks, smoked during pregnancy.	1:241728236-249240000 (1q43-q44) De novo	Dup	**Pathogenic**. Macrocephaly, ID, prominent forehead, micrognathia/retrognathia.^21^
							X:1-50350659 (Xp22.33-p11.22) *De novo*	Del	**Pathogenic**. Turner Syndrome. Xp22 deletion previously reported in CP.^10^
150432P Duo	F	D	2	41 + 3	BW: 19th percentile. ASD, toe walker, DD. CNV events in this individual result from a de novo unbalanced translocation	Hypotension, induced vaginal delivery, no complications	2:1-2550499 (2p25.3) De novo	Del	**Pathogenic**. ID, obesity, ASD, ADHD, delayed psychomotor development.^19^ Previously reported in CP.^10^
							20:54945507-62960000 (20q13.2-q13.33) De novo	Dup	**Pathogenic**. *Role in CP uncertain*. DD, cardiac malformation, clinodactyly of 5^th^ finger, receding chin, protruding upper lip, large low set ears, upslanting palpebral fissures, epicanthus, microphthalmia, high scalp.^37^
157439P Trio	M	D	3	29 + 2	BW: < 1 percentile. Epilepsy, DD, cleft lip and palate, ASD, twin to twin transfusion syndrome (donor), IUGR with NICU stay. Twin brother with the same variant has milder but similar symptoms (GMFCS 1)	Fibromyalgia syndrome, fetal distress, elective caesarean delivery without onset of labour	3:33759248-34277749 (3p22.3) De novo	Del	**Likely pathogenic**. This locus includes *PDCD6IP*. Support from knockout mouse model^28^ and zebrafish in this study.
143425P Trio	M	RH		38 + 3	BW: 13^th^ percentile. Epilepsy, stroke in utero	History of miscarriage and stillbirths, *giardia* infection during pregnancy, spontaneous vaginal delivery, fetal distress	16:29474649-30200303 (16p11.2-p12.2) De novo	Del	**Pathogenic**. ASD, Speech anomalies, hypotonia with hyporeflexia, altered agility, seizures, macrocephaly, Chiari I malformation.^26^
192475P Duo	M	RH	3	36 + 0	BW: 77th percentile. DD, bilateral talipes, abnormal arch and branching of the heart, polymicrogyria, T-cell immunodeficiency, mandibular osteomyelitis, laryngomalacia, NICU stay	History of miscarriages, premature labour by spontaneous vaginal delivery	22:18655870-21480623 (22q11.21) De novo (inferred)	Del	**Pathogenic**. 22q11.2 deletion syndrome, congenital heart disease, immunodeficiency, palatal abnormalities, hypocalcaemia and ID.^23^ Previously reported in CP.^9^
183465P Trio	F	D	3	34 + 0	BW: 66th percentile. Twin 1 with vanishing twin syndrome for twin 2 (gender not stated), NICU stay	Placental abruption due to clotting disorder causing bleeding in second half of pregnancy, emergency caesarean after onset of labour	22:18656485-21463730 (22q11) De novo	Dup	**Pathogenic**. 22q11.2 duplication syndrome. ID, behavioural disorder, delayed psychomotor development, hearing impairment, seizures and heart abnormalities.^25^ Previously reported in CP^11^
165447P Duo	M	Dy		38 + 4	BW: 63rd percentile. Perinatal stroke, NICU stay	Hypertension, induced vaginal delivery, no complications	2:54893010-55200400 (2p16.1) *Unknown*	Dup	**Uncertain**. This locus harbours the brain-expressed *EML6* gene.
187469P Duo	F	Dy	5	37	BW: 16th percentile. Signs of ASD, not formally diagnosed, DD, anxiety. Previously identified de novo balanced translocation 46,X,t(X;1)(q13.1; q32.1)	Maternal diabetes, maternal hypertension, anemia, emergency caesarean	2:106498314-107074142 (2q12.2) *Maternal*	Dup	**Uncertain**. Similar duplications in DECIPHER have unrelated phenotypes. No known disease genes in this interval.
105114P Trio	F	LH		38	BW: 59th percentile. MRI at 9 m: Right MCA territory encephalomalacia, consistent with previous infarct, low protein C level	Gastric infection during pregnancy, spontaneous vaginal delivery, tight cord	2:125668980-127806247 (2q14.3) *Maternal*	Del	**Uncertain**. No similar deletions in DECIPHER. Down regulation of GYPC is associated with hereditary elliptocytosis.
129411P Trio	F	Q	5	27	BW: 5th percentile, IUGR	Smoked during pregnancy, major bleeding, breech, emergency caesarean	3:9831395-9908996 (3p25.3) *Paternal*	Dup	**Uncertain**. No similar duplications in DECIPHER. No known disease genes in this interval.
108117P Trio	M	LH	1	39	BW: 66th percentile. Borderline ID, epilepsy, anxiety, left homonymous hemianopia, MRI 3d, 5 y and 7 y: Right MCA territory infarction and extensive encephalomalacia involving the central regions, frontal, parietal and occipital lobes, NICU stay	Fetal distress, spontaneous vaginal delivery	5:76989059-77563443 (5q14.1) *Maternal*	Dup	**Uncertain**. No similar duplications in DECIPHER. *AP3B1* (Hermansky-Pudlak Syndrome OMIM: 608233).
164446P Duo	F	D	2	27 + 0	BW: 30th percentile. Hyperbilirubinemia, hypertonia, respiratory distress syndrome, NICU stay	Preterm infection, history of miscarriages and preterm births incompetent cervix, threatened miscarriage, preterm labour spontaneous vaginal delivery	6:17282979-17987418 (6p22.3) *Maternal*	Dup	**Uncertain**. *This locus has potential for involvement in CP*. Six individuals in DECIPHER with similar sized duplications (260928, 270332, 270990, 287970, 295582 and 331142). Global DD, behavioural disorder, microcephaly, short stature and ID.
132414P Trio	M	H	1	40 + 1	BW: 92nd percentile. Epilepsy, von Willebrand disease, NICU stay	Urinary condition and threatened miscarriage, depression, fetal distress emergency caesarean after onset of labour	9:1056659-2923296 (9p24.3-p24.2) De novo	Dup	**Uncertain**. *This locus has potential for involvement in CP*. Homozygous loss of function mutations in *VLDLR* cause cerebellar ataxia, intellectual disability and disequilibrium syndrome [OMIM: (CAMRQ1) 224050]
101110P Single	M	SH	2	29	BW: 98th percentile. Plagiocephaly, ASD, PVL, DD, neonatal seizures, obesity, right convergent squint, previous central apnoea, double hernia (39 w), twin 2 with female twin 1 deceased in utero, NICU stay	History of infertility, polycystic ovarian syndrome, gestational diabetes, hypertension, obstetrical bleeding for entire pregnancy, preterm labour, fetal distress, growth restricted emergency caesarean.	9:131733020-132481734 (9q34.11) *Unknown*	Dup	**Uncertain**. *This locus has potential for involvement in CP*. Two individuals in DECIPHER with similar sized overlapping duplications (256548 and 340614); both with ID. One individual (340614) had delayed fine and gross motor development.
89098P Trio	F	SD	3	28 + 3	BW: 7th percentile. PVL, IUGR, NICU stay	Chorioamnionitis, funisitis, GBS, preterm labour emergency caesarean delivery	10:73822516-74987970 (10q22.1-q22.2) De novo	Del	**Uncertain**. *This locus has potential for involvement in CP*. *ASCC1* [OMIM: 614215] rare autosomal recessive congenital myopathy with similarity to spinal muscular atrophy.^38^ *MICU1* [OMIM: 605084] recessive myopathy with motor and speech delay, progressive proximal muscle weakness and learning difficulties.^39^
49049P Trio	M	RH	1	35	BW: 5^th^ percentile. MRI at 3 y: Unilateral periventricular grey matter heterotopia, with two subependymal nodules on the lateral wall of the right lateral ventricle, IUGR, low protein C level, blood clotting issues, NICU stay	Spontaneous vaginal delivery	14:35593381-35871320 (14q13.2) *Paternal*	Dup	**Uncertain**. A SNP (rs1048990) that results in up regulation of *PSMA6* may be associated with increased risk of myocardial infarction.^40^
180462P Duo	F	D	4	40	BW: 27th percentile. Holoprosencephaly, diabetes insipidus, GERD, bilateral cleft lip and palate, epilepsy, global DD, hearing loss	Milroy’s disease, fever during pregnancy, threatened labour at 32 weeks, spontaneous vaginal delivery	16:2906033-3071871 (16p13.3) *Unknown*	Del	**Uncertain**. No similar duplications in DECIPHER. Does not overlap *CREBBP*
104113P Trio	M	Dy, Q	4	40 + 2	BW: 31st percentile. NE secondary to intrapartum anaphylaxis, neonatal seizures, hypotonia in all 4 limbs, DD, short stature, gastronomy fed, undescended testes, kyphosis, sialorrhea with drooling, nose bleeds, NICU stay	Threatened miscarriage emergency caesarean after onset of labour.	19:2076893-2901121 (19p13.3) De novo	Del	**Uncertain**. *This locus has potential for involvement in CP*. Two similar individuals in DECIPHER (269826 and 287279): Macrocephaly, DD, generalized hypotonia, abnormal dentition, bone abnormalities, ectodermal dysplasia, ID, pachygyria
46046P Duo	F	D,Dy	2	39	BW: 40th percentile. Ultra sound 2d: Global cerebral oedema. MRI at 8d: bilateral infarction of frontal and parietal lobes, dyspraxia, neonatal seizures, in special class	Maternal hypertension, gastric infection during pregnancy, emergency caesarean, fetal distress	20:47711417-47989806 (20q13.13) *Maternal*	Dup	**Uncertain**. Two individuals in DECIPHER with similar duplications: 265056 (delayed puberty, growth hormone deficiency, short stature) and 341719 (specific learning disability)
120402P Trio	M	D	4	26 + 3	BW: 16th percentile. Epilepsy, twin 2 (gender of twin 1 unknown), NICU stay	Preterm labour, spontaneous vaginal delivery	X:99663478-100290718 (Xq22.1) *X-linked*	Dup	**Uncertain**. *This locus has potential for involvement in CP*. Five individuals with X-linked or unknown inheritance in DECIPHER (258232, 278836, 291903, 326486, 337860). ID, ASD, aplasia or hypoplasia of the cerebellar vermis, microcephaly, strabismus, hypermetropia, hypotonia, seizures.

*F* female, *M* male, *S* spastic, *D* diplegia, *R/L/H* right/left/hemiplegia, *Q* quadriplegia, *Dy* dyskinesia, *ASD* autism spectrum disorder, *ADHD* attention deficit hyperactivity disorder, *BW* birth weight, *d/m/y* days/months/ years, *DD* developmental delay, *GBS* group B *Streptococcus* infection, *ID* intellectual disability, *IUGR* intrauterine growth restriction, *MCA* middle cerebral artery, *NE* neonatal encephalopathy, *NICU* neonatal intensive care unit, *PVL* periventricular leukomalacia

Individual validation of CNV with CytoSNP-850 arrays on 14 individuals, including 150432P and 181463P with the translocations, supported six out of seven deletions and all nine duplications with positive detection calls. The deletion, which was not detected with PennCNV, was in 98098P chr10:73822516–74987970 (all intervals are mapped to hg19 coordinates) (Table S18). Further investigation of the array data of all 14 individuals revealed 23 duplications and five deletions that included known genes (based on GENCODE v28 annotations) that were not identified by CoNIFER (Table S24). All of these CNV were of uncertain clinical significance and all except one were found in single genes.

We next sought to determine if the expression of genes in, or adjacent to all loci detected by CoNIFER were affected by these CNV. We used existing RNA-Seq data generated from RNA extracted from lymphoblastoid cell lines (LCL) of these individuals.^15^ In 22 out of the 23 CNV loci identified, we determined a trend for significant differential expression of most, but not all expressed genes, as defined by Z-scores of greater than 2 or less than −2 (Tables S1–S23). The direction of differential gene expression (up or down) was highly correlated (Pearson *r* = 0.75) with the copy number gain or loss respectively (Fig. S2, Tables S1–S23). No significant deviation in gene expression was determined in sample 165447P (dup chr2:54893010-55200400), however only one gene in this interval had detectable expression in the RNA-Seq data (Table S10). Of note, sample 98098P that did not have their deletion validated by the SNP chip, had a consistent trend for significant down regulation of the expression of all 11 genes detected in that interval (Table S18), suggesting that it was a bona fide deletion.

**Fig. 1:**
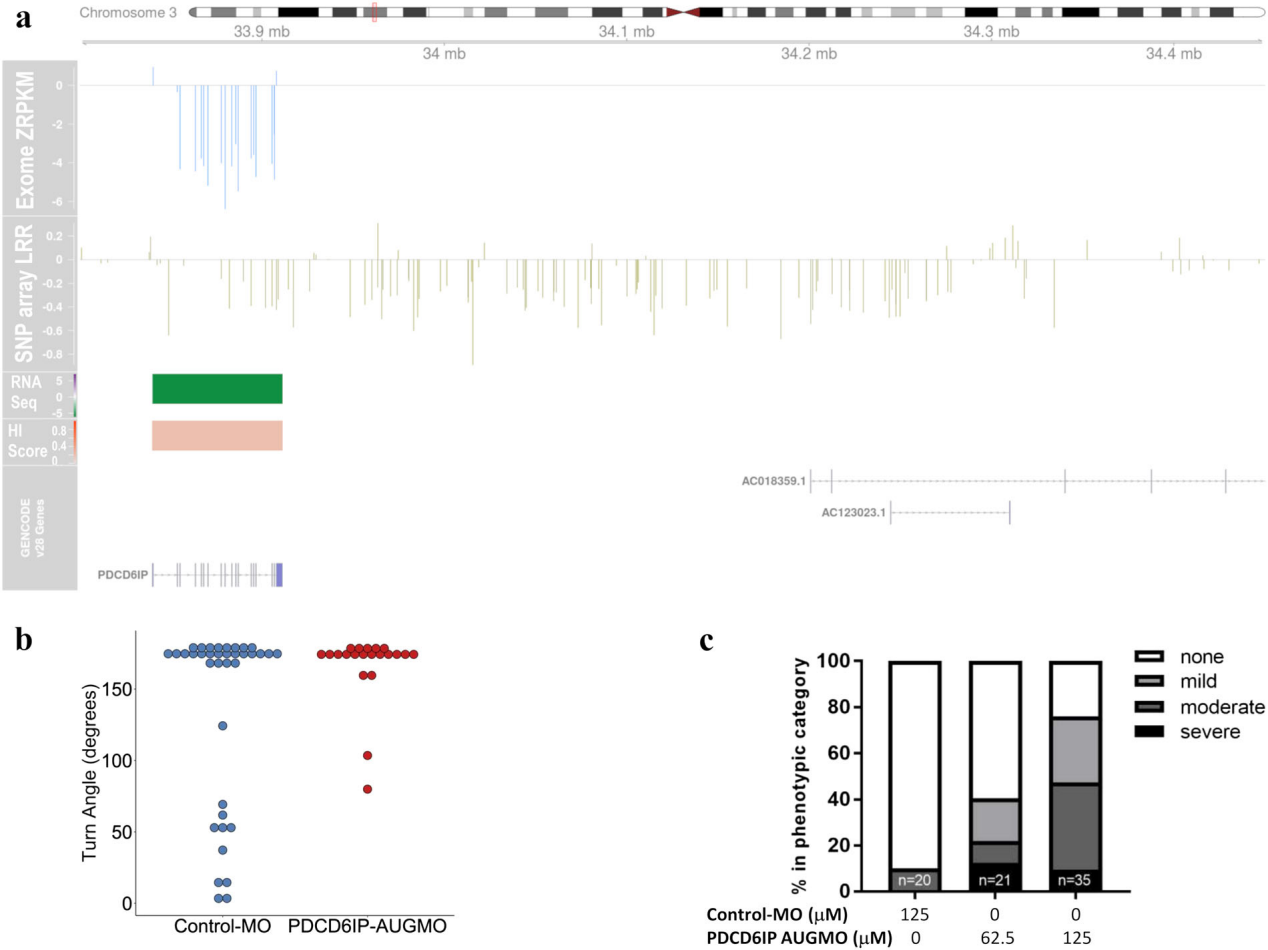
*PDCD6IP* deletion detected by CoNIFER with confirmed effects on gene expression and support for pathogenicity from a zebrafish knock-down model defines a new gene for CP. **a** Composite plot showing genomic context of the *PDCD6IP* deletion. The ideogram for chromosome 3 at the top of the plot shows the genomic location of the CNV boxed in red. Base pair positions of the CNV from the hg19 build of the human genome are shown below the ideogram. Data from CoNIFER (Exome SVD-ZRPKM values) are shown in blue with the zero baseline indicated by a grey line. PennCNV Log R ratios are shown in khaki with the zero baseline indicated by a grey line. RNA Seq outlier gene expression for each gene in its genomic context is plotted as a heat map; purple indicates upregulation and green indicates down regulation, grey or light shading indicates the gene is below the cut off of −2 to 2. Haploinsufficency percentile scores (HI scores) from DECIPHER are shown as a heatmap with genes with the darkest orange the most sensitive to copy number variation. GENCODE v28 gene models are shown at the bottom of the plot. **b** Average turning angle of zebra fish injected with the PDCD6IP-AUGMO at concentrations of 62.5 μM (red, *n* = 21) compared to a control injected group (blue, *n* = 37) over three trials stimulating movement with a tap in the first two trials and light in the final trial. PDCD6IP-AUGMO treated fish moved with significantly greater turning angles, *p* < 0.05 Student’s two-tailed *T*-test assuming equal variances. **c** Quantification of phenotypes with increasing concentration of the PDCD6IP-AUGMO. Larvae were classified as having mild, moderate or severe developmental abnormalities depending on whether they had one, two to three, or more than four of the following phenotypes: loss of pigment in eye, decreased eye size, grey matter hindbrain, small head, domed cranium, cardiac abnormality, decreased body size and tail curvature or kinking

Among the potentially pathogenic CNV we detected, there was one locus found in individual 157439P and his similarly though more mildly affected monozygotic twin brother in which a deletion down-regulated the expression of only one gene: *PDCD6IP* (Table 1 and Fig. 1a). Both brothers had bilateral cleft lip and palate and both had seizures with age of onset at 3 years. The seizures later resolved (earlier in the mildly affected twin). Movement was affected in both brothers manifesting as quadriplegia in the most significantly affected brother and mild quadriplegia in the mildly affected brother. Computed tomography (CT) scan of the more severely affected brother at age 3 years showed features consistent with periventricular leukomalacia (PVL) as predicted from early postnatal ultrasound scans. The more mildly affected brother had a MRI scan at age 8 which also revealed features consistent with PVL (also predicted by early postnatal ultrasound scan) and some thinning of the corpus callosum. The *PDCD6IP* gene has not been previously implicated in any neurodevelopmental disorder and presented a unique opportunity from this study to assess the likely pathogenicity of this deletion in an animal model. We knocked down expression of the corresponding zebrafish ortholog (*pdcd6ip*) using both translation blocking (PDCD6IP AUGMO) and splice blocking (PDCD6IP SBMO) morpholinos. We observed significant effects on zebrafish movement that included spontaneous, hyperactive and erratic swimming behaviour (Video S1 and S2). There was a significant (*p* < 0.05) increase in the maximum turning angle of fish treated with PDCD6IP AUGMO compared to controls (Fig. 1b). Video evidence showed that this was because the PDCD6IP AUGMO were unable to swim in a straight line, suggesting that motor control was impeded (Video S1 and S2). There were also consistent morphological defects such as microcephaly and cardiac oedema that were specific and concentration dependent to the PDCD6IP AUGMO and to a lesser extent the PDCD6IP SBMO compared to the control (Fig. 1c and Fig. S3).

In combination, our results identified pathogenic and likely pathogenic CNV in at least a further 7 (3.7% [95% CI, 1.8–7.6%]) out of an unselected cohort of 186 individuals with CP (Table 1). The total fraction of individuals with pathogenic and likely pathogenic genetic variants in this unselected CP cohort (including 14% detected by WES^8^ and a further 5% of SNVs by RNA Seq^15^) is 22.7%.

## Discussion

CNV have long been known to be significant contributors to the aetiology of neurodevelopmental disorders.^17^ Similar to other neurodevelopmental disorders such as epilepsy, ID or ASD, CNV contribute to the pathogenesis of CP.^9–12^ For the purpose of molecular diagnosis, chromosomal microarrays (with SNP and/or oligonucleotide probes) are usually selected for detection of CNV because of their high sensitivity and specificity. In this study, we elected to take advantage of our unique, existing exome sequencing data of 186 individuals with CP to make an efficient and cost-effective survey of CNV burden in the largest unselected CP cohort to date, rather than reanalyse the entire cohort with arrays. The proportion of this cohort with pathogenic and likely pathogenic CNV in CP was lower compared with a previous study of a similar unselected cohort which could be due in part to the lower resolution of detection of exome CNV analysis compared to arrays.^10^ It is interesting to note that in our previous CNV study of 50 cases that were also reanalysed in this study (finding no additional CNV) that we did not find any de novo CNV. It is possible that the resolutions of the arrays we used previously, as well as the resolution for detection by exomes has contributed to this and smaller de novo CNV may yet be found.

RNA-Seq data identified genes within or adjacent to each CNV that were affected in expression and in one case, this confirmed a suspected deletion that was detected by the exome but not by SNP array. Based on the genotype tissue expression project (GTEx) median tissue expression data, 84.8% of transcripts expressed in cortex are also expressed in LCL (transcripts per million reads, TPM > 0) and expression levels are highly correlated, Pearson *r* = 0.83 therefore, this approach is useful for assessing the effects of CNV encompassing genes expressed in both tissues even in the context of neurodevelopmental disorders.^18^

### Pathogenic CNV loci implicated in CP

We identified deletions of 2p25.3, Xp and 22q11.21 that overlap with deletions identified in previous studies of CNV in CP (Table 1).^9–11^ Deletions of 2p25.3 [OMIM: 616521] encompass a syndrome of ID, obesity, ASD, attention deficit hyperactivity disorder (ADHD) and delayed psychomotor development.^19^ The neurological phenotypes associated with 2p25.3 deletions have been linked with haploinsufficiency of *MYT1L* and that gene was also deleted in the individual in this study, however it was not expressed in LCL^19^ (Table S4).

Full or partial monosomy of the X chromosome in females causes Turner Syndrome. While not reported frequently, impaired movement that is not associated with intelligence scores has been observed in Turner Syndrome.^20^ There are two previously reported females with CP attributed to partial chromosome X monosomy.^10,11^ In this study, we discovered an individual with Xp monosomy and a terminal 1q43q44 trisomy due to translocation t(X;1), (p11.22; q43) (Table 1; Tables S2 and S3). This individual had a complex phenotype with dysmorphic features, short stature, microcephaly, global developmental delay and CP. By 11 years 4 months she was wheelchair bound. A review of 112 deletions and 47 duplications encompassing 1q43q44 noted problems with muscle tone and muscle control in 27 of the 47 individuals with pure or complex duplications.^21^ We concluded that, this translocation was pathogenic and there may be contributions from both the deleted Xp and the duplicated 1q43q44 to CP in this case.

Both deletions and duplications of 22q11.2 have been previously reported in CP.^9,11^

22q11.2 deletion syndrome [OMIM: 192430], also known as velocardiofacial or DiGeorge syndrome is clinically heterogeneous and the most common, recurrent, pathogenic microdeletion that occurs in humans.^22^ CP and other movement phenotypes are infrequently reported in this multisystemic disorder that affects the heart, immune system, parathyroid, craniofacial and central nervous system development.^23^ In one report, mild left hemiparesis was noted in a male with 22q11.2 deletion and cerebellar atrophy on brain MRI.^24^ Duplications of 22q11.2 are reported less frequently than deletions [OMIM: 608363] and penetrance is variable. A clinical review of multiple individuals affected by this duplication found that motor delay and hypotonia were frequent features.^25^

### Pathogenic loci not previously implicated in CP

We identified two de novo CNV that are known to be pathogenic for neurodevelopmental disorders, also with incomplete penetrance and variable expressivity; a 16p11.2-p12.2 deletion [OMIM: 611913]^26^ and a 1q21.1 duplication. A review of clinical observations in 136 individuals with 16p11.2 deletions noted abnormal agility in 50%^26^ while duplications of 1q21.1 overlapping with the individual in this study have abnormal agility in 39%.^27^ While the known pathogenic CNV loci described above are associated with distinct neurodevelopmental syndromes, our clinical and genetic data, combined with previous observations of significant effects on movement, strongly suggest them as causing CP in these individuals.

At the time of writing, there were 33 individuals in the DECIPHER (DatabasE of genomiC varIation and Phenotype in Humans using Ensembl Resources) database with open access phenotype information that includes the term “cerebral palsy”. We did not find any CNV or SNV in that cohort that overlapped with new CNV reported in this study.

### Novel loci and genes

A de novo microdeletion at 3p22.3 encompassing only the gene for programmed cell death 6 interacting protein *PDCD6IP* (also commonly referred to as *ALIX*) that segregated with CP in monozygotic twin brothers was associated with significant down regulation of that gene in LCL derived from one of the affected individuals (Fig. 1a, Table S6). PDCD6IP has critical roles in cytokinesis and also interacts with the endosomal sorting complex required for transport (ESCRT) multiprotein complexes which, in turn are required for trafficking of multi vesicular bodies in the cell. Mice null for *Pdcd6ip* develop hydrocephalus resulting in bilateral enlargement of the lateral ventricles, thinning of the cerebral cortex and atrophy of the hippocampus.^28^ The loss of *Pdcd6ip* caused disorganisation of alignment of cilia and a loss of integrity of epithelial cell barriers in the brain and other tissues.^28^ These mouse data combined with our observations from zebrafish strongly implicate *PDCD6IP* as a disease locus in neurodevelopmental disorders.

### Variants of uncertain significance

We identified five deletions and nine duplications that passed our filtering criteria and occurred at loci that are not yet implicated in CP or other neurodevelopmental syndromes. Based on available clinical data and functional annotation of the genes within these regions, we predict that six of these events have potential to be involved in CP (Table 1). We singled out these six CNV found on 6p22.3, 9p24.3-p24.2, 9q34.1, 10q22.1-q22.2, 19p13.3 and Xq22.1 (Table 1 and Tables S15-S18, S21 and S23) because in each case they have either multiple reported individuals in DECIPHER with similar but not identical phenotypes and/or encompass genes known to be involved in delayed motor development. Noteworthy among these CNV, was a duplication we identified located within Xq22.1 and inherited from an unaffected mother (Table 1, Table S23). Within this X-linked duplication, the mRNA polyadenylation factor *CSTF2* was found to be significantly up regulated (Table S23) while other genes with detectable levels of expression were unaffected. This male also had a male second cousin related through possible female carriers that was also affected by CP, however a DNA sample was not available for segregation testing. Individuals with similar duplications in this region in DECIPHER have phenotypes consistent with those in the individual in this study including ID, ASD and epilepsy (Table 1).

For each of these variants of uncertain significance, additional patients and further cell molecular and animal modelling will be required to confirm or deny their pathogenicity.

## Conclusions

**Fig. 2:**
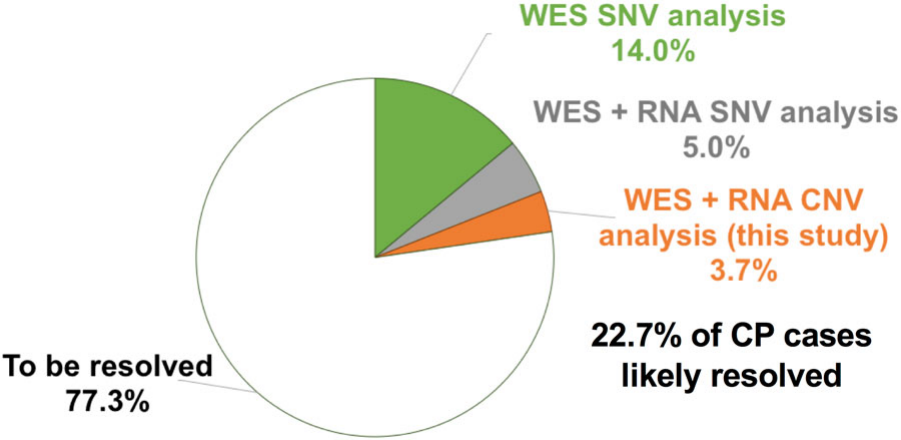
Overview of pathogenic variants detected in three studies in an unselected Australian cohort of 191 individuals living with CP. Green slice shows percent of cases resolved by our original WES study.^8^ Grey slice summarizes SNVs (premature terminations codons and splice variants) that affect gene expression.^15^ Orange slice summarizes findings from this study

**Fig. 3:**
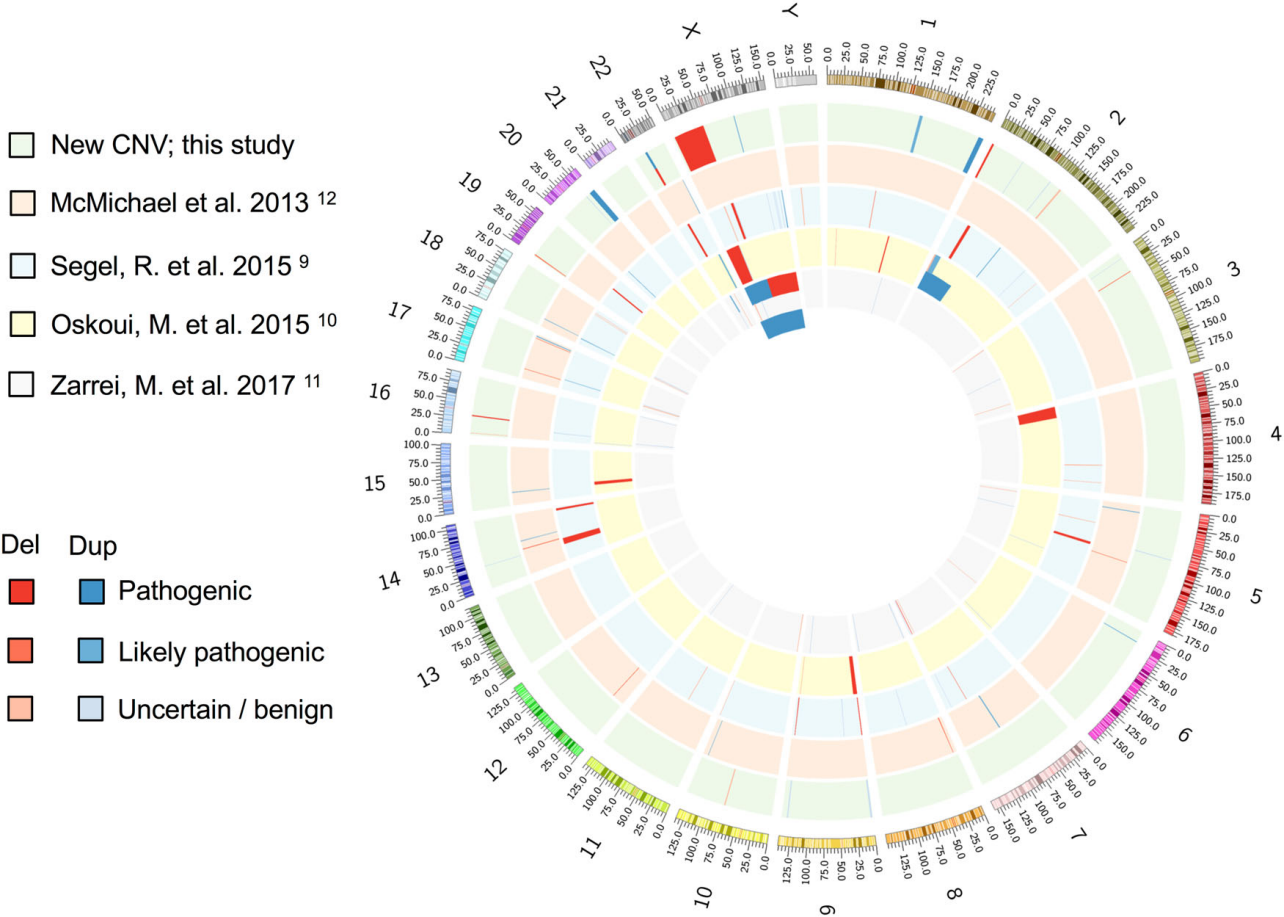
CNV reported in CP to date. Circos plot generated using the ClicO FS tool shows the human karyogram on the outer edge with deletions in red and duplications in blue from this study and the previous four reports as indicated by the study key. The saturation of colour of each CNV indicates its likelihood for pathogenicity

Through CNV analysis of exome data and additional integration of RNA-Seq we have resolved at least 7/186 additional individuals accounting for 3.7% this cohort. In combination with SNV identified in our previous analyses, we have shown a pick up rate of potentially pathogenic variants in an unselected CP cohort of 22.7% (95% CI, 15.6–31.8%), (Fig. 2).^8,15^ With this study, we have expanded on the known genetic determinants of CP and identified both new and recurrent CNV loci that confer significant risk for CP (Fig. 3). The genomic landscape of CNV in CP shows potential hot spots on chromosomes 2, 22 and X, and highlights considerable genetic heterogeneity that underlies the clinical heterogeneity of CP (Fig. 3). This anecdotal evidence for enriched loci implicated in CP suggests a meta-analysis combining all 450 individuals with CNV data to date with additional cases yet to be analysed through the newly formed International CP Genomics Consortium would be warranted.^29^ Despite considerable genetic heterogeneity of CP, the number of individuals now identified with pathogenic variants to date suggests genetic testing with chromosomal microarray and if available, clinical exome sequencing should be considered early in the diagnosis. There are good prospects for further improvement of diagnostic pick up rates by employing whole genome sequencing, which will increase the resolution of detection of structural variants as well as coding and non-coding SNV. We have shown that integrated analysis of paired exome and RNA-Seq data combined with follow up animal model analyses aids in the identification and functional and diagnostic interpretation of novel pathogenic variants in CP.

## Methods

### Exome sequence data

This study was approved by the Women’s and Children’s Health Network Human Research Ethics Committee (HREC) and informed written consent was obtained from all participants. A flowchart giving an overview of this study is provided in Fig. S4. We started with a set of 472 exome BAM files from 191 unrelated and unselected individuals living with CP and their parents and siblings where available (1 family with multiple affected individuals, 98 parent-proband trios, 72 parent-proband duos and 20 singletons).^8^ The unselected cohort had the following profile: 45:55% female to male ratio, 48% were premature (less than 37 weeks), 22% had intrauterine growth restriction (i.e. a birth weight below the 10th percentile relative to gestational age), 55% had at least one neurodevelopmental co-morbidity (epilepsy, ASD, developmental delay or ID) and gross motor function classification scores (GMFCS) ranged from 1 to 5 with median of 2. The profile is similar to that of the Australian CP registry report suggesting minimal selection bias.^30^ For additional information on selection criteria for CP diagnosis of this cohort, please refer to our previous study.^8^ We used Picard tools *CollectHsMetrics* and found 12 samples (two trios and three duos) had low coverage of less than 80% of targets covered at 20× or more and less than 30× mean and median coverage of targets; these were removed from the study (Fig. S5A and S5B). We used the *vcftools relatedness2* algorithm to confirm family relationships were as described in the original study and found evidence of two sample mix ups within these data that were corrected for subsequent analyses.^31^ The results of the original study were not altered by these mix ups. The final cohort included 186 unrelated individuals with CP.

### Exome CNV analysis

Copy number detection in exome data was carried out using CoNIFER scripts.^16^ We used the SeqCap EZ HGSC VCRome V2.1 (Roche NimbleGen) target file (covering 45.2 Mbp of sequence) as the array template. A singular value decomposition (SVD) cut off score of 7 was used (Fig. S6). We removed CNV that overlapped by 80% or greater with CNV that had minor allele frequencies (MAF) greater than or equal to 0.01 from studies with more than 40 individuals in the DECIPHER common CNV database (DECIPHER v9.5). CNV that were recurrent in more than ten individuals (i.e. four or more trios, or MAF > 0.02) in the cohort were also removed because we considered these unlikely to be pathogenic due to their high frequency. De novo inheritance of deletions in incomplete trios was inferred by examining the remaining haplotype compared to the available parental sample.

### SNP array validation

Validation of CNV regions identified by CoNIFER was carried out using Illumina Infinium CytoSNP 850K arrays. Library preparation, hybridisation, scanning and data acquisition were performed according to the manufacturer’s protocols, as a service by the Australian Genome Research Facility. Detection of CNV calls was carried out with *PennCNV* using default parameters for autosomal and X-linked analyses respectively.^32^

### Gene expression outlier analysis

RNA-Seq data corresponding to 182/186 CP cases in this study and 20 unrelated control samples were generated as part of a separate and dedicated gene expression analysis study for CP.^15^ We incorporated this expression data resource into a new analysis to determine the influence of CNV identified in this study on gene expression. Briefly, RNA was extracted from Epstein Barr Virus transformed LCL using QIAGEN RNeasy kits, as per the manufacturer’s protocol. Library preparation and RNA-Seq were performed as a service by the UCLA Neuroscience Genomics Core Facility. The TruSeq v2 kit (Illumina) was used to generate un-stranded libraries with 150 bp mean fragment sizes and 50 bp pair end sequencing performed using the HiSeq2500 (Illumina). Sequence data were mapped to Illumina iGenomes hg19 build of the human genome using tophat2.^33^ Read counts were generated with *htseq-count*.^34^ Z-statistics for each gene in each sample were calculated over all 202 samples using the *scale* function from the R statistical programming language v3.4.4.

### Correlation and visualization of CNV and gene expression data

Exome ZRPKM values from CoNIFER and gene expression *Z*-scores from scaled RNA-Seq data were correlated in each sample by matching values in each matrix to their location in the genome. These data consisted of 11,507 genes expressed in LCL with matching exome coverage. Loci in which there was at least one pair of correlated gene expression and exome CNV Z-scores less than −2 or >2 were prioritized for further analysis. Combined exome ZRPKM, array log R ratio and outlier gene expression *Z*-scores were plotted in their genomic contexts using the *Gviz* package in Bioconductor (Tables S1–S23).^35^

### Zebrafish husbandry and morpholino injections

Zebrafish studies were approved by the University of Adelaide Animal ethics committee. *Danio rerio* were bred and maintained at 28 °C on a 14-h light//10-h dark cycle. Embryos were collected from natural mating of the Tübingen strain (Tu), grown in embryo medium (E3), and staged.^36^ Translation blocking (PDCD6IP AUGMO 5'-ACGGGACAGAAATAAACGTCGCCAT-3'), splice blocking (PDCD6IP SBMO 5′-TCTTCAGGCCAATGTCTTACCGCAG-3′) and control (5′-CCTCTTACCTCAGTTACAATTTATA-3′) morpholinos were synthesized by Gene Tools LLC (Corvallis, OR, USA). Fertilized zebrafish embryos were rinsed in E3 medium and injected at the one cell stage. Embryos were always injected with individual morpholinos in solution at total concentrations of 250, 125 or 62.5 μM depending on the assay. At 6 h post fertilization, any embryos that were unfertilised were discarded.

### Activity analysis using the DanioVision observation chamber

Larval activity was tracked at 4 days post fertilisation (dpf) using the DanioVision Observation Chamber (Noldus) which is fitted with a Basler GenICam camera, independent light source, temperature control unit (set to 28 °C) and tapping device. The settings used for tracking experiments were defined using EthoVisionXT software. Trials were run in triplicate and whole wells where tracking failed during the experiment were excluded. Experiments were performed with the researcher blind to the identity of the injected morpholino.

### Imaging

Embryos were examined at 24hpf and any embryos which had failed to develop were removed. Larvae were examined at 4dpf and scored blind for morphological abnormalities and touch-evoked escape response. Larvae were categorised as phenotypically normal or having mild, moderate or severe abnormalities. Larvae were imaged after activity assays using a Nikon SMZ1000 dissecting microscope and micrographs taken using a Leica DFC450 C camera and Leica Application Suite software.

### Statistics

Binomial CIs were calculated for all proportions using the Wilson method implemented by the *binom* package in R. Differences in movement measurements between zebrafish injected with control and PDCD6IP morpholinos were tested using Student’s two-tailed *T*-test assuming equal variances with corrections for multiple comparisons made using the Bonferroni method.

### Web Resources

DECIPHER: https://decipher.sanger.ac.uk/

OMIM: http://omim.org

## Supplementary information

Supplemental Data

Data Set 1

Control morpholino

pdcd6ip AUGMO

## Data Availability

The data generated or used during this work are available subject to compliance with our obligations under human research ethics from the corresponding author upon reasonable request.

## **Supplementary information**

**Supplementary information** accompanies the paper on the *npj Genomic Medicine* website (10.1038/s41525-018-0073-4).
