# Author Correction: Pathogenic copy number variants that affect gene expression contribute to genomic burden in cerebral palsy

**DOI:** 10.1038/s41525-019-0086-7

**Published:** 2019-05-31

**Authors:** Mark A. Corbett, Clare L. van Eyk, Dani L. Webber, Stephen J. Bent, Morgan Newman, Kelly Harper, Jesia G. Berry, Dimitar N. Azmanov, Karen J. Woodward, Alison E. Gardner, Jennie Slee, Luís A. Pérez-Jurado, Alastair H. MacLennan, Jozef Gecz

**Affiliations:** 1 Robinson Research Institute & Adelaide Medical School, University of Adelaide, Adelaide, South Australia 5000 Australia; 2 Data61, Commonwealth Scientific and Industrial Research Organisation, Ecosciences Precinct, Dutton Park, Brisbane, QLD 4102 Australia; 3 School of Biological Sciences, University of Adelaide, Adelaide, South Australia 5005 Australia; 4 Department of Diagnostic Genomics, Queen Elizabeth II Medical Centre, PathWest, Nedlands, WA 6009 Australia; 5 School of Biomedical Sciences, University of Western Australia, Perth, WA 6009 Australia; 6 Genetic Services of Western Australia, King Edward Memorial Hospital, Subiaco, WA 6008 Australia; 7 Genetics Unit, Universitat Pompeu Fabra, Barcelona, 08003 Spain; 8 Hospital del Mar Research Institute (IMIM) and Centro de Investigación Biomédica en Red de Enfermedades Raras (CIBERER), Barcelona, 08003 Spain; 9 SA Clinical Genetics, Women’s and Children’s Hospital & University of Adelaide, Adelaide, South Australia 5006 Australia; 10 South Australian Health and Medical Research Institute, Adelaide, South Australia 5000 Australia

**Keywords:** Next-generation sequencing, Neurodevelopmental disorders, Gene expression, Structural variation

**Correction to:** *npj Genomic Medicine* 10.1038/s41525-018-0073-4, published online 14 December 2018

In the original published version of this Article, in the Methods section, the morpholino sequence for PDCD6IP AUGMO sequence was incorrectly stated as 5'-CCTTCTGACCGCTGCATGGTTTCTC-3'. It should have been 5'-ACGGGACAGAAATAAACGTCGCCAT-3'. This has been corrected for the HTML and PDF versions of the Article.

