# Supplementary material for: Pathogenic copy number variants that affect gene expression contribute to genomic burden in cerebral palsy
Source: NPJ Genom Med. 2018 Dec 14;3:33. doi: 10.1038/s41525-018-0073-4 (PMC6294788; doi:10.1038/s41525-018-0073-4)
Supplement: Supplementary file 1 — Supplemental Data [file 41525_2018_73_MOESM1_ESM.pdf]

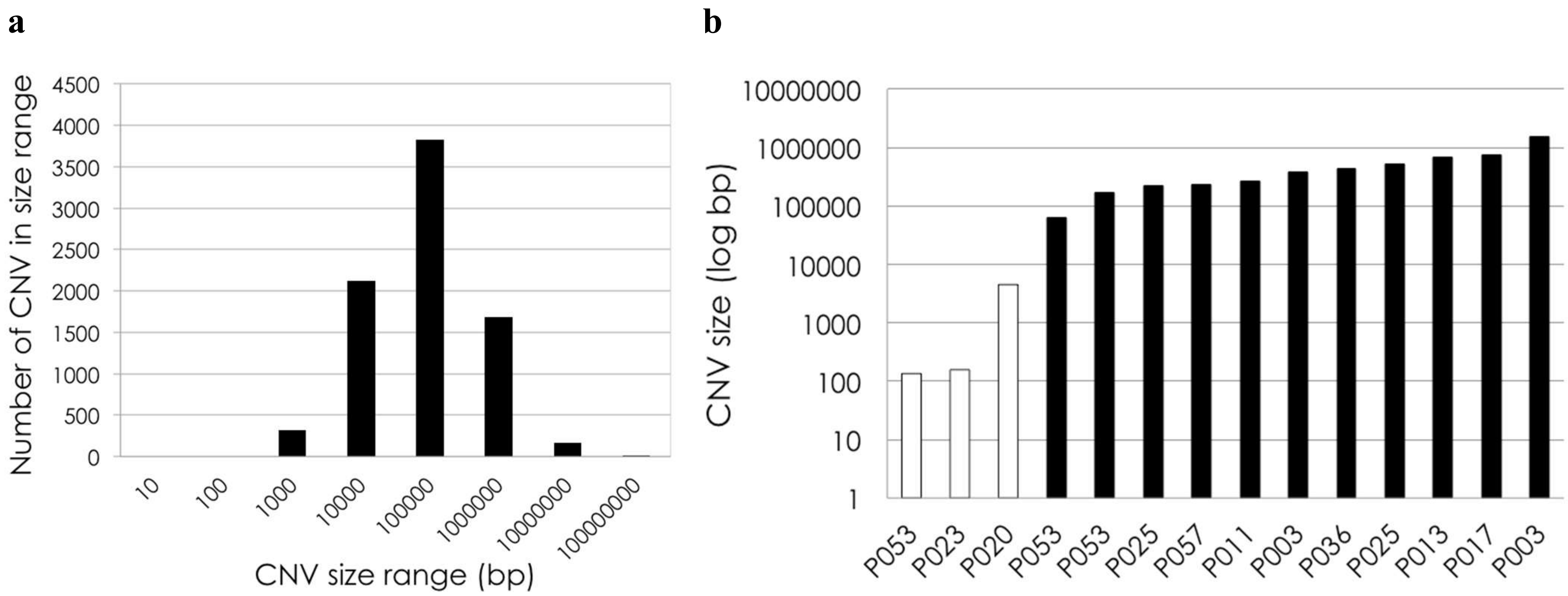

Figure S1. Sensitivity and specificity of detection. **a**. Plot of size ranges of CNV detected by CoNIFER. **b**. Plot of previously identified CNV[12] detected (black bars) or not detected (white bars) by CoNIFER.

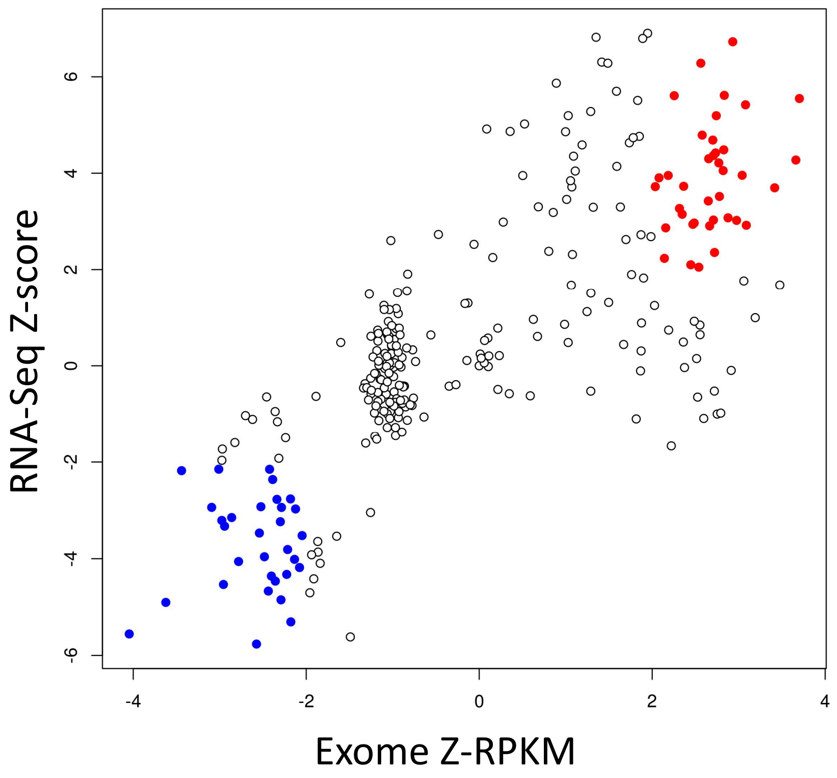

Figure S2. Gene dosage and RNA expression are highly correlated. Plot shows correlation (Pearson r = 0.75) between gene dosage as measured by average exome Z-RPKM for each gene compared to the RNA-Seq Z-score for that gene of all CNV loci shown in Table 1. Blue and red colouring indicate genes where Z-scores for both RNA-Seq and exome data are less than -2 or greater than 2 respectively.

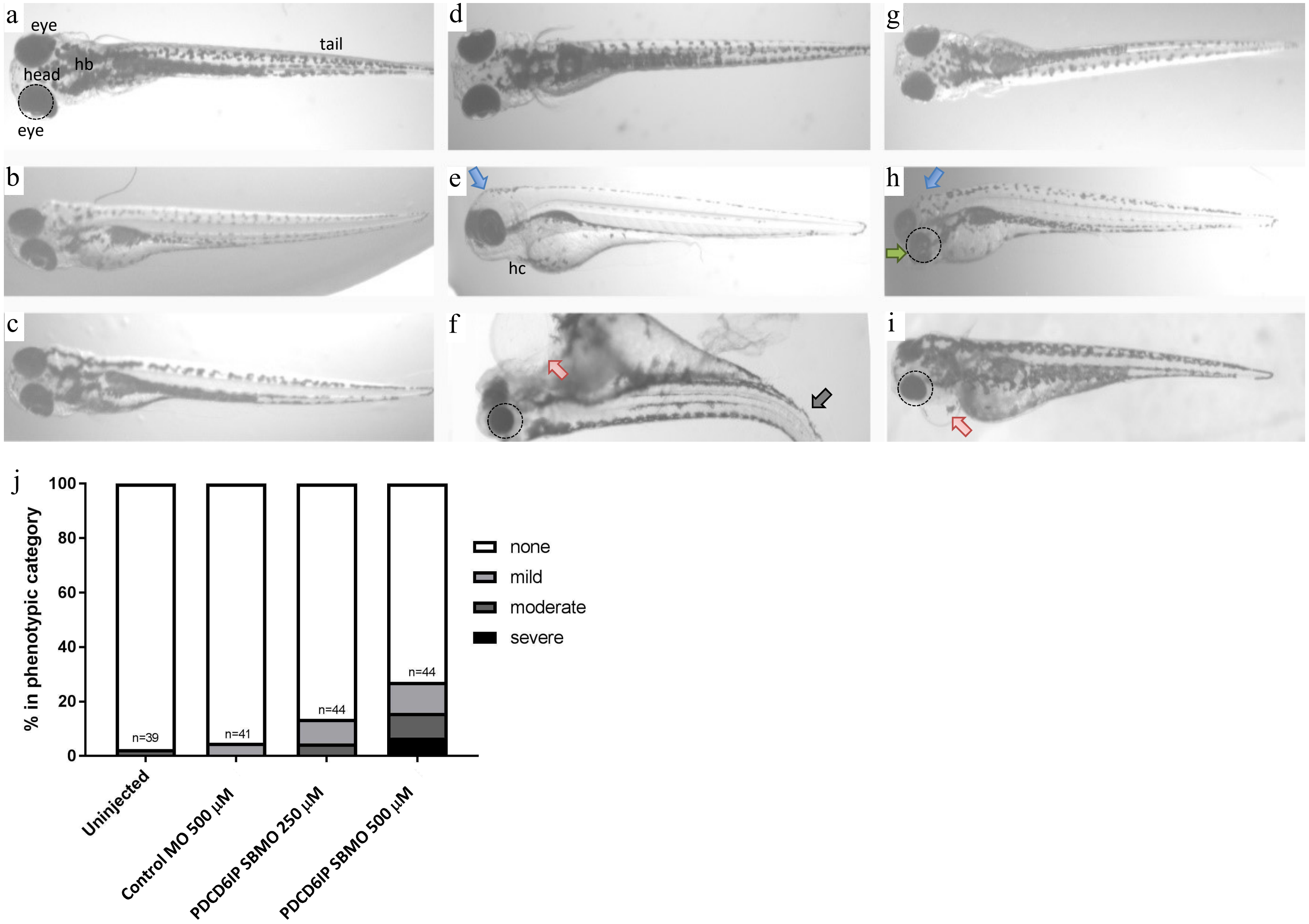

Figure S3. PDCD6IP morphants display abnormal brain and heart development. **a-c:** The majority of larvae in the injected control morpholino group had no obvious phenotype. Normal anatomy of the eye, head, hindbrain (hb) tail and heart chamber (hc) are shown (**a**). Mild generalized loss of pigment in normal sized eyes was observed at low frequency in the injected control morpholino group. **d-f:** Representative images showing the phenotypic range in larvae injected with 125 µM of PDCD6IP AUGMO. **g-i:** Representative images showing the phenotypic range in larvae injected with 62.5 µM of PDCD6IP AUGMO. **j.** Larvae injected with PDCD6IP splice blocking morpholino (PDCD6IP SBMO) were classified as having mild, moderate or severe developmental abnormalities depending on whether they had one, two to three, or more than four of the following phenotypes: loss of pigment in eye (green arrow **h**), decreased eye size (dashed circle **h,f,i**), grey matter hindbrain (**e,f**), small head (**h,f,i**), domed cranium (blue arrow **e,h**), cardiac abnormality (red arrow **f,i**), decreased size (**h,f,i**) and tail curvature (grey arrow **f**).

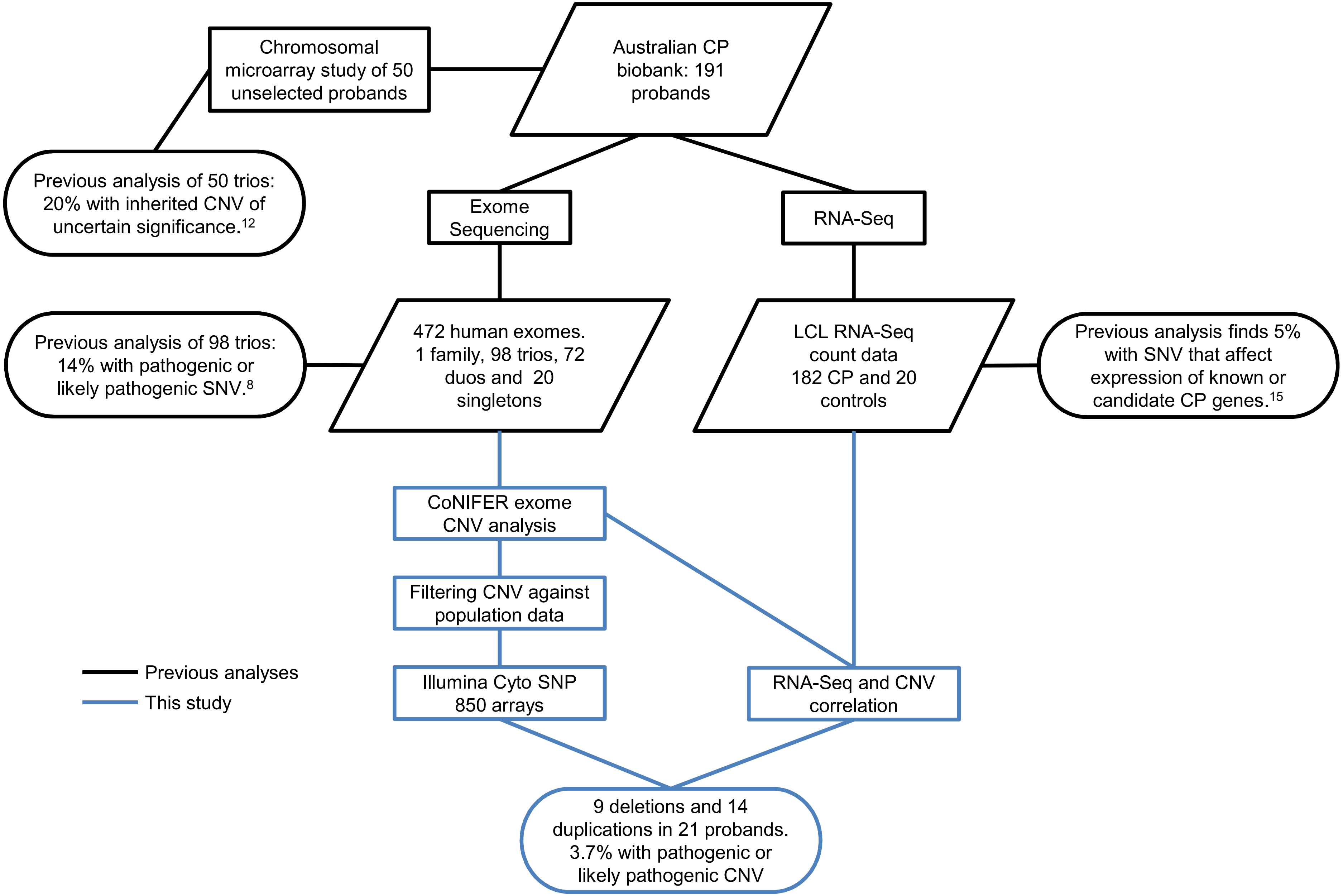

Figure S4. Flowchart of the experimental design. Flow chart shows the extent of analyses carried out on the Australian CP biobank. An unselected cohort of 191 individuals with CP and their parents and family where available. Copy number variant (CNV), single nucleotide variant (SNV), Lymphoblastiod cell line (LCL)

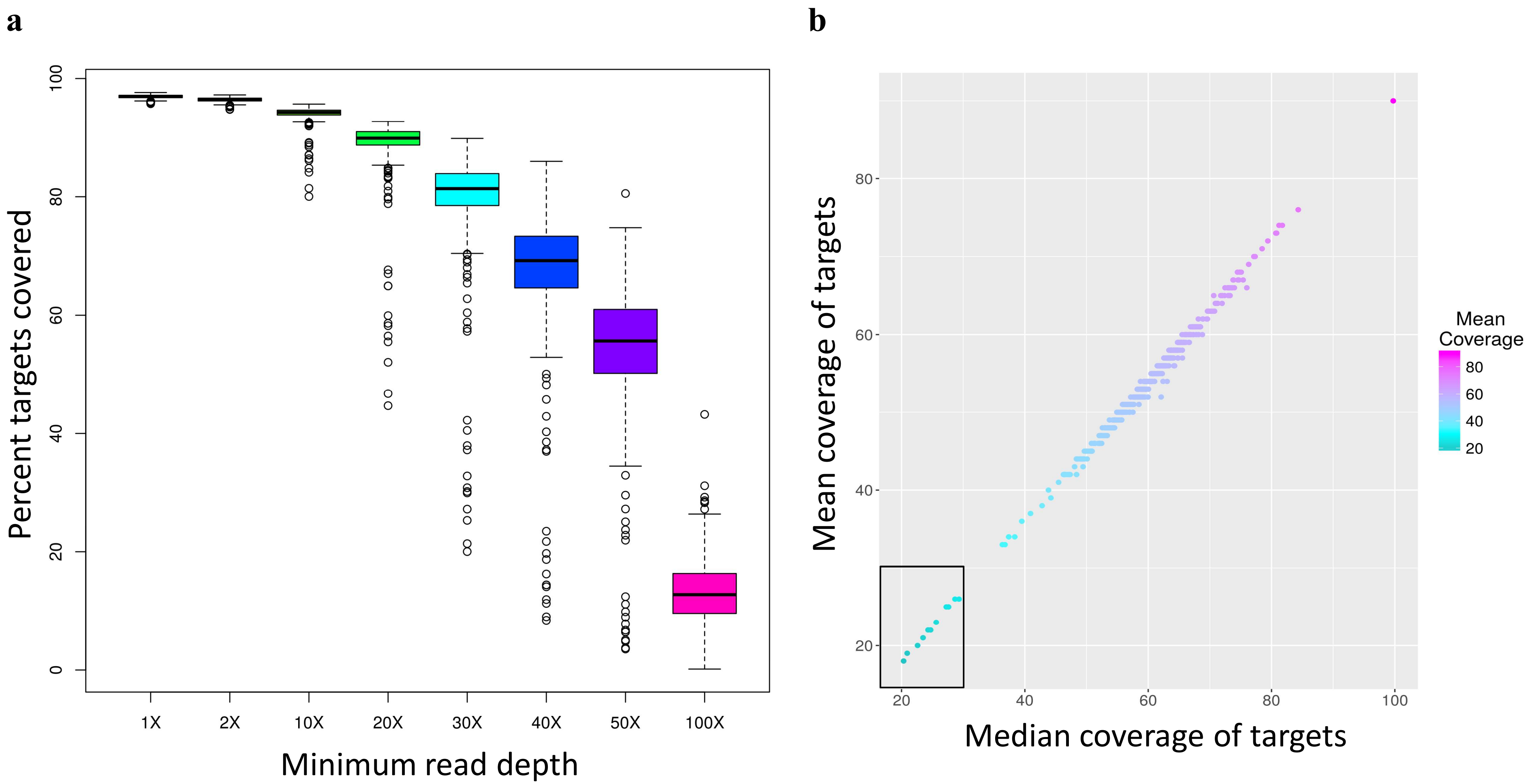

Figure S5. Exome quality checks. **a**. boxplot of coverage levels compared to percent of the VCRome V2 exome target coverage, over 75% of samples had better than 30 fold coverage of at least 80% of targets. **b**. Plot of mean vs. median coverage shows slightly higher median coverage values per exome. Boxed area indicates samples with less that 30x mean coverage that were removed from these analyses.

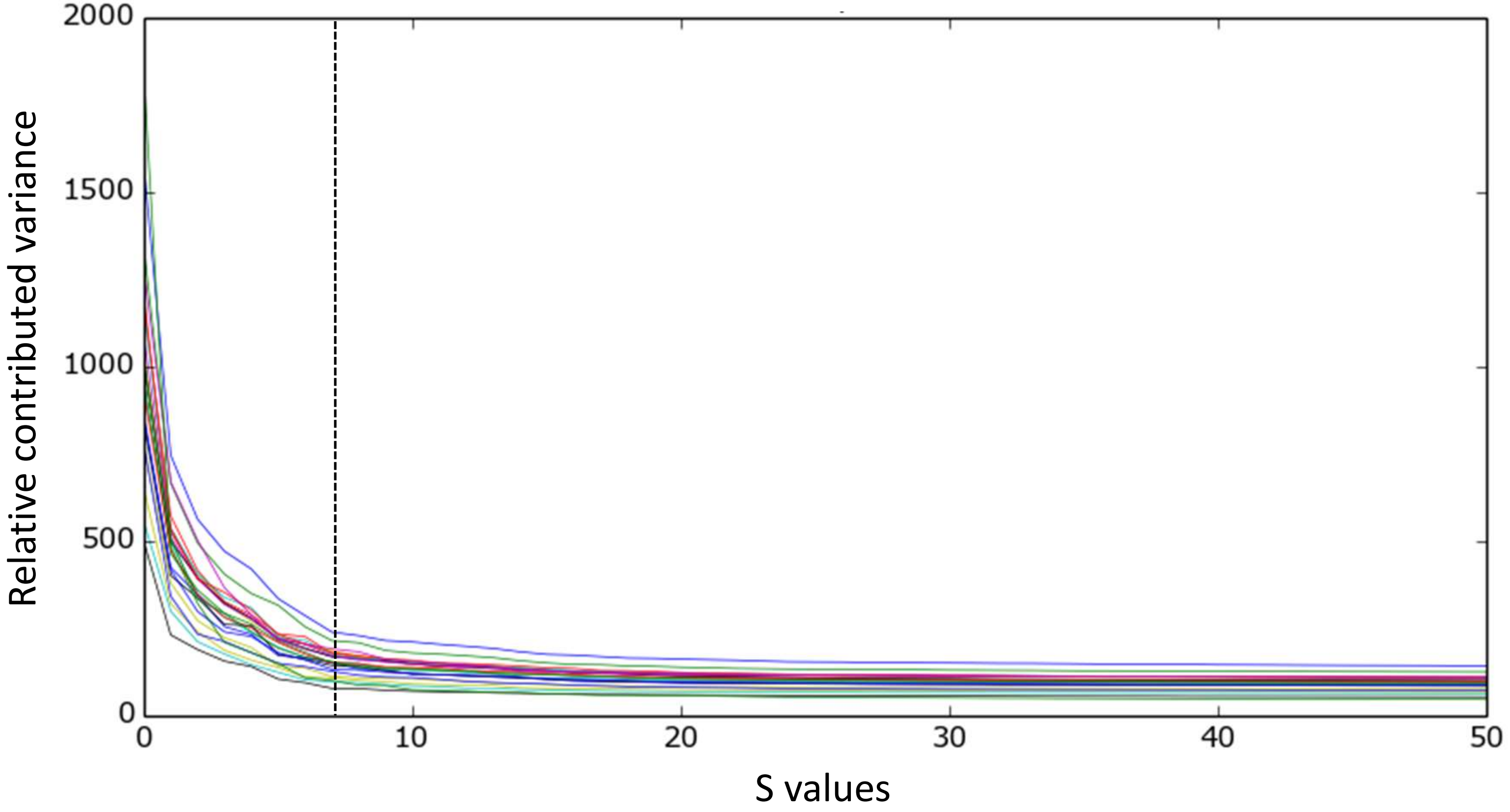

Figure S6. Representative plot of first 50 singular (S) for values as calculated by CoNIFER. The relative contributed variance of all CNV is expected to be minor therefore the S values that contribute the majority of relative variance to the initially calculated exome copy number Z-scores can be removed and the Z-scores recalculated based on this. A cut off of 7 was used in this study indicated by the dotted line on the plot which lies at the inflection point in this plot therefore removing the majority of unwanted variance.

Table S24. Additional CNV identified from Illumina CytoSNP 850K arrays

Sample	chr	start	end	startSNP	endSNP	numSNP	length	Type	Genes	RNA-Seq Z-score	pLI	OMIM	Brain or LCL Expression	Pathogenicity prediction and known phenotypes
101110P	chr2	140992793	141017744	rs1038851	kgp10486793	13	24,952	Dup	*LRP1B*		0.51		Brain	Uncertain. No disease association. Knock out mouse normal
101110P	chr4	86986237	86998661	rs59198932	kgp2418608	8	12,425	Dup	*MAPK10*		0.3	602897	Brain	Uncertain. Translocations associated with epilepsy, ID and delayed psychomotor development
101110P	chr5	24537481	24593325	rs6882202	rs16893586	20	55,845	Dup	*CDH10*		0.33		Brain	Uncertain. No disease association
101110P	chr11	66789861	66816507	rs7930695	rs3741190	8	26,647	Del	*SYT12*		0		Brain	Uncertain. No disease association
120402P	chr2	61235855	61278696	rs1182688	kgp14631098	26	42,842	Del	*PEX13*	-1.499	0	601789	Brain, LCL	Uncertain. Associated with Zellweger syndrome.
120402P	chr2	140988796	141006794	rs12463968	kgp24622640	12	17,999	Dup	*LRP1B*		0.51		Brain	Uncertain. No disease association. Knock out mouse normal
120402P	chr2	189564693	189629741	rs10173369	rs13004707	14	65,049	Dup	*DIRC1*				Neither	Likely benign. No disease association
120402P	chr6	102315257	102348065	kgp10227050	kgp12405634	14	32,809	Dup	*GRIK2*		1	138244	Brain	Uncertain. Mental retardation, autosomal recessive 6
120402P	chr8	113959206	113975571	rs11990766	rs10505197	4	16,366	Dup	*CSMD3*		0.82		Brain	Uncertain. No disease association
120402P	chr11	16250267	16259625	rs12284156	rs1949480	23	9,359	Dup	*SOX6*		1	607257	Brain	Uncertain. Potential involvement in skeletal development
120402P	chr12	21291358	21319996	rs4149016	rs7973837	35	28,639	Dup	*SLCO1B1*		0		Neither	Likely benign. No disease association
120402P	chr12	21350687	21355537	rs4149062	rs11045859	15	4,851	Dup	*SLCO1B1*		0		Neither	Likely benign. No disease association
132414P	chr1	98292912	98307395	rs17117249	kgp6051196	9	14,484	Dup	*DPYD*	1.072	0	612779	Brain (low), LCL	Uncertain. Dihydropyrimidine dehydrogenase deficiency
132414P	chr8	113302023	113387333	rs929686	rs11785419	10	85,311	Dup	*CSMD3*		0.82		Brain	Uncertain. No disease association
132414P	chr17	73863929	73888806	rs2034947	rs9916659	9	24,878	Del	*TRIM47, TRIM65*	NA, 0.546	0		Brain, LCL (*TRIM65*)	Uncertain. Supported by homozygosity of exome variants. This locus associated with white matter hyperintensities
143425P	chr3	93742842	93772810	rs12629037	kgp18070011	14	29,969	Dup	*ARL13B*	-0.099	0	608922	Brain, LCL	Uncertain. Joubert syndrome 8
143425P	chr18	30528021	30556985	rs11662235	rs7238802	4	28,965	Dup	*CCDC178*		0		Neither	Likely benign. No disease association
157439P	chr4	91917416	92009492	rs2035116	rs10023583	24	92,077	Dup	*CCSER1*		0		Brain	Uncertain. No disease association
157439P	chr6	102337555	102343504	exm568572	rs17784776	5	5,950	Dup	*GRIK2*		1	138244	Brain	Uncertain. Mental retardation, autosomal recessive 6
157439P	chr21	22848121	22867087	rs17003875	rs1380591	8	18,967	Dup	*NCAM2*		0.21		Brain	Uncertain. No disease association
164446P	chr4	167652786	167661584	rs11943562	rs7678726	6	8,799	Dup	*SPOCK3*		0.06		Brain	Uncertain. No disease association
165447P	chr2	189972818	189979778	rs62184182	kgp10876808	11	6,961	Dup	*COL5A2*		1	120190	Brain (low)	Uncertain. Ehlers-Danlos Syndrome
165447P	chr13	70388448	70419939	rs2325234	rs2911506	6	31,492	Dup	*KLHL1*		0		Brain	Uncertain. No disease association
181463P	chr4	70713066	70716627	rs2055533	rs1220715	13	3,562	Dup	*SULT1E1*		0		Neither	Likely benign. No disease association
181463P	chr14	37966740	38018045	kgp11131101	kgp19704777	22	51,306	Dup	*MIPOL1*		0	606850	Brain (low)	Uncertain. Polydactyly
181463P	chr22	37603021	37607981	rs2071710	rs9610669	4	4,961	Del	*SSTR3*	0.850	0		Brain, LCL	Uncertain. Supported by homozygosity of exome variants. No disease association
183465P	chr19	46268902	46273135	rs2341097	rs3745804	16	4,234	Del	*SIX5*	1.327	0.01	600963	Brain (low), LCL	Uncertain. Branchiorenal syndrome 2
192475P	chr4	70713066	70716627	rs2055533	rs1220715	13	3,562	Dup	*SULT1E1*		0		Neither	Likely benign. No disease association

Abbreviations: chr: chromosome, Dup: duplication, Del: deletion, LCL: lymphoblastoid cell line
